# Partial seizure due to COVID19 infection in an infant 

**Published:** 2020

**Authors:** Alireza NATEGHIAN, Saeed ANVARI

**Affiliations:** 1 Department of Pediatrics, Aliasghar children hospital, Iran University of Medical Science, Tehran, Iran; 2 Department of Pediatrics, Division of Pediatric Neurology, Milad Hospital, Social Security Organisation, Tehran, Iran

**Keywords:** COVID19, children, partial seizure

## Abstract

We live at the time of the coronavirus pandemic in the world (1, 2). The symptoms of COVID19 are similar in children and adults. However, children with confirmed COVID19 have generally shown mild symptoms (3). The symptoms in children include cold-like symptoms, such as fever, runny nose, and cough, vomiting, and diarrhea. In this study, we describe an eight-month-old boy with recurrent partial seizure and mild diarrhea. It was later revealed that he was COVID19 positive.

## Introduction

Coronavirus can cause neurological symptoms in patients. An analysis in china has shown that children younger than 10 years account for only 1% of COVID-19 cases (4), similar to the proportion for SARS-CoV and MERS-CoV epidemics (5, 6, 7). Neurological manifestations in adults include acute cerebrovascular disease, impaired consciousness, and skeletal muscle injury (8). However, in children, especially infants, there are no specific reports of neurological complications. In this study, we report an eight-month-old boy with recurrent partial seizure and mild diarrhea and COVID19 PCR positive.

## Case report

Our case was an eight-month-old boy with mild diarrhea and abnormal movement. He was taken to Erfan Niayesh Hospital. Diarrhea started three days before hospitalization, and the patient had a mild cough with diarrhea. One day after the beginning of diarrhea, the patient had a seizure several times. The seizures were on waking state and in the form of tilting of the lips and blinking and clonic movements of the limbs for a few seconds. During the four hours, the seizures were similarly repeated seven times. The patient did not lose consciousness during the seizure attacks, but, at the end of the seizures, he fell asleep. I would like to inform you that the patient did not have a fever and a runny nose. He was born from nonrelative parents, with birth

weight 3250 gr., weight 9500gr, head circumference equal to 37cm, cesarean section. Parents have had three pregnancies and one abortion, and one healthy baby. He had no major or important problem at birth. Also, he did not have icter at birth. In family history, his father had a seizure at the age of eighteen years. old without any other problem. Meanwhile, the patient’s mother had a scattered cough about three weeks ago. He had normal physical examination and development. The infant could roll over and move forward slightly. The environmental connection was appropriate with age.

First analytical studies showed WBC=8700 (neutrophil=27% and lymphocyte=68%),

Hb=12.7, MCV=80.04 plt=346000, ESR=29, Ca=11.5, Mg=2, Blood sugar=82 , CRP=2,

ALT=30, AST=46, Na=138, K=4.7, BUN=8, Cr=0.5, TSH=5.4, NH3=83, Lactate=15, Urine

Reducing Substance=Negative, CT scan of the lungs were normal

And finally: COVID19 PCR=Target detected Seizures did not recur after phenobarbital was started. The patient was treated for coronavirus infection.

The patient was scheduled to be discharged on an outpatient basis for a brain MRI and EEG(after the quarantine period is over). The patient had no significant problems at the time of discharge.

## In conclusion

COVID19 can infect the nervous system and skeletal muscle as well as the respiratory tract in adults; however, in infants, it may cause seizures. The disease information should be gradually collected to get a definitive view of its neurological problems in children and infants
